# The dynamics of mortality in follow-up time after an acute myocardial infarction, lower extremity arterial disease and ischemic stroke

**DOI:** 10.1186/1471-2261-10-57

**Published:** 2010-11-25

**Authors:** Ilonca Vaartjes, Ineke van Dis, Diederick E Grobbee, Michiel L Bots

**Affiliations:** 1 Julius Center for Health Sciences and Primary Care, University Medical Center Utrecht, Utrecht, the Netherlands; 2 Netherlands Heart Foundation, The Hague, the Netherlands

## Abstract

**Background:**

Most studies providing data on survival in patients with atherosclerosis only address a single disease site: heart, brain or legs. Therefore, our objective was to determine risk of death after first hospital admission for atherosclerotic disease located at different sites.

**Methods:**

A nationwide cohort of patients hospitalized for the first time for acute myocardial infarction (AMI), peripheral arterial disease of the lower extremities (PAD) or ischemic stroke was identified through linkage of national registers. The mortality rate in AMI patients was compared to mortality rate in ischemic stroke and PAD patients by estimating relative risks (with 95%CI). Cox's proportional hazard models were used to estimate sex differences in risk of death.

**Results:**

Case fatality was high for ischemic stroke patients (men:21.0%, women:23.8%) and AMI patients (men:12.7%, women:20.9%) though low for PAD patients (men:2.4%, women:3.5%). The five-year risk of death was similar for male AMI compared to PAD patients (men: RR1.04; 95%CI 0.98-1.11). The risk of death for ischemic stroke patients remained the highest though the differences with AMI and PAD patients attenuated.

**Conclusions:**

The dynamics of mortality over follow-up time clearly differ between atherosclerotic diseases, located at different vascular beds. The risk of death increases considerably over follow-up time for PAD patients, and 5 years after first hospital admission the differences in risks of death between AMI- and PAD patients and between AMI- and ischemic stroke patients have largely attenuated. Clinicians should be aware of these dynamics of mortality over follow-up time to provide optimal secondary prevention treatment.

## Background

Atherosclerosis is a systemic disease leading to various symptoms and clinical events. Acute myocardial infarction (AMI), ischemic stroke and peripheral arterial disease of the lower extremities (PAD) are common atherosclerotic manifestations, by which the coronary arteries, carotid arteries and peripheral arteries of the lower extremities respectively, play a major role. In recent decades, clinical and population-based studies have provided extensive data on the prognosis of patients diagnosed with atherosclerosis [1]. These data primarily address the prognosis of one manifestation of atherosclerotic disease rather than different manifestations of atherosclerosis [2,3]. Several studies have reported associations of cardiovascular risk factors with progression of atherosclerosis [4,5], although the association of the risk factors with progression of atherosclerosis is inconsistent across different vascular beds [6]. This might suggest that prognosis of patients diagnosed with atherosclerosis may depend on the location of the vascular bed where atherosclerosis predominantly becomes manifest.

In a previous study, where we used parts of the data from the present study, we investigated long-term prognosis of PAD and compared mortality among first hospitalized PAD patients with mortality among patients first hospitalized for stroke and AMI. We reported that five-year death rate was somewhat higher among stroke patients and almost similar for PAD and AMI patients [7]. Caro et al. [8] investigated potential risk factors for PAD mortality and presented long term survival among PAD patients together with survival among patients suffering from stroke and AMI. They reported that the crude five-year death rate among patients diagnosed with PAD was lower than death rates among patients diagnosed with stroke, but higher than death rates among patients diagnosed with AMI. These studies suggest that there may be a different prognosis per manifestations of atherosclerosis. However, these studies did not include short-term prognosis [7] or did not provide age-and sex-specific mortality [8], despite the evidence that mortality depends on age and gender. The purpose of the present study, therefore, was to estimate both detailed short-term and long-term mortality risks, stratified by age and gender, in a large, nationwide cohort of patients first hospitalized for AMI, ischemic stroke and PAD.

## Methods

For the present study, cohorts were drawn from 1995, 1997 and 2000. The choice of years was pragmatic at the time the project was initiated in 2001. The total population of the Netherlands in 1995, 1997 and 2000 was 15,424,122 (men 7,627,428, women 7,796,640), 15,567,107 (men 7,696,803, women 7,870,304) and 15,863,950 (men: 7,846,317, women: 8,017, 633), respectively. Approximately 14% of the population was older than 65 years. To construct a cohort of patients admitted for the first time due to ischemic stroke, AMI and PAD, information from the national Hospital Discharge Register (HDR) and the Population Register (PR) were linked. Information on cause of death has been derived from the Cause of Death Register of Statistics Netherlands. The registers and linkage procedures have been described in detail previously for a cohort of acute myocardial infarction [9] and stroke patients [10].

### Cohort enrolment

All hospital admissions for acute myocardial infarction (AMI) between January 1^st ^and December 31^st ^1995, (ICD-9-CM code 410, ICD-10-CM code I21) and all hospital admissions for ischemic stroke (ICD-9-CM code 434, 436, ICD-10-CM code I63) and peripheral arterial disease of the lower extremities (PAD) (ICD-9-CM code 443.9, ICD-10-CM code I73.9) between January 1^st ^and December 31^st ^1997 and January 1^st ^and December 31^st ^2000 respectively, were selected. No information on severity of disease was available. Therefore, we assumed that PAD patients diagnosed with ICD-9 code 443.9 (peripheral vascular disease unspecified, intermittent claudication not otherwise specified (NOS). Peripheral: angiopathy NOS, vascular disease NOS, Spasm of artery. Excluded are atherosclerosis of the arteries of the extremities and spasm of cerebral artery) represents patients hospitalized with moderate/severe claudication, ischemic rest pain and ulceration or gangrene (Fontaine stage IIb, III and IV respectively), as these patients have an indication for surgical interventions [11].

By merging with the PR, only those patients with a unique combination (linkage variables date of birth, sex and numerical part of postal code) were selected. Next, the first hospital admissions in 1995 for AMI and in 1997 and 2000 for ischemic stroke and PAD were selected. To identify first admissions, the file was merged with a file in which, through the same linkage procedures, information was collected on hospital admissions that occurred previously (1991-1995, 1995-1997 and 1995-2000 respectively) for the same condition. For the years 1991-1994 a different - and slightly less reliable - linkage procedure was used, because the digital PR only started in 1995 [12]. Those with a previous admission for the same condition were excluded, for example, those with a previous hospitalization for cerebrovascular accident (CVA) were excluded from the ischemic stroke cohort, but not from the cohorts dealing with PAD or AMI. This approach left a cohort of patients admitted to hospital for the first time because of the occurrence of ischemic stroke, PAD or AMI. Finally, the cohort was linked with the Cause of Death Register to obtain information on date and cause of death (in-hospital and out-of-hospital) during follow-up. Patients were censored if they migrated out of the Netherlands during follow-up.

### Comorbidity

The presence of comorbidity (cardiovascular diseases (ICD-9-CM codes 390-459) or diabetes mellitus (ICD-9-CM code 250)) was determined on the basis of the discharge diagnosis of previous hospital admissions or on the basis of a secondary diagnosis at the moment of the index admission.

No information on risk factors (hypertension, smoking) or medication use was available in the register.

### Data analysis

Analyses were performed for AMI, ischemic stroke and PAD. Survival time was calculated as the time from the initial admission date in 1995 for AMI and 1997 or 2000 for ischemic stroke and PAD, to the date of death from any cause, or to the date that a patient was censored, whichever came first. The 28-day case-fatality, 1-year and long-term (5-year) mortality was computed by age and gender according to the actuarial life table method and expressed as percentages. To make results comparable with other community-based studies, the overall rates were adjusted to the European standard population [13]. The 1-year and long-term (5-year) mortality was also computed for patients who survive for at least 28 days after hospital admission. Cox regression models were used to study gender differences in mortality at 28 days, 1 year and 5 years. For each period, a model was used to adjust for age. Results were presented as hazard ratios (with 95%CI). The mortality rate in AMI patients was compared to the mortality rate in ischemic stroke patients and PAD patients by calculating relative risks (with 95%CI). Data were analyzed with SPSS software, version 14.0 (SPSS Inc, Chicago, Illinois, USA).

Approval for the use of the anonymized patient data was covered by a general agreement between Statistics Netherlands and Dutch Hospital Data (DHD). Additionally, the Dutch association of hospitals (NVZ) and Dutch federation of University Hospitals (NFU) approved the use of the hospital registration data for this study. No separate ethical approval was necessary for the use of these data. All analyses were performed in accordance with privacy legislation in the Netherlands [14].

## Results

**Figure 1:**
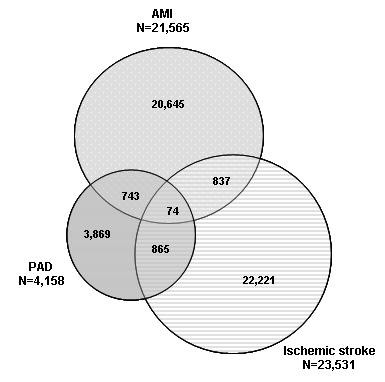
**Diagram of patients first hospitalized for acute myocardial infarction (AMI), ischemic stroke, or peripheral arterial disease of the lower extremities (PAD)**. Number of patients diagnosed with atherosclerosis at single or multiple sites of vascular bed.

More men than women were hospitalized for first AMI (14,463 men, 7,102 women) in 1995 and PAD (2,539 men, 1,619 women) in 1997 and 2000, while more women than men were hospitalized for first ischemic stroke (11,381 men, 12,150 women) in 1997 and 2000. General characteristics are provided in Table 1. In all three groups women were older than men. Men had more previous admissions for ischemic heart disease than women. Women were known to have diabetes mellitus more often than man. More men were admitted to academic hospitals than women. This difference was the most pronounced among PAD patients (men: 11.2%, women: 7.8%). The length of stay was longer for women than men. De mean length of stay was clearly the longest among ischemic stroke patients. The largest part of the patients (93% of PAD patients, 94% of ischemic stroke patients and 95% of AMI patients) were diagnosed with an atherosclerotic manifestation at one location of the vascular bed (Figure 1). These patients had not been previously admitted for AMI, PAD and ischemic stroke.

**Table 1:** Characteristics of patients with a first hospitalization in the Netherlands for acute myocardial infarction (AMI), peripheral arterial disease of the lower extremities (PAD) and ischemic stroke

	AMI#		PAD†		Ischemic stroke‡	
	Men	Women	Men	Women	Men	Women
Number of patients	14,463	7,102	2,539	1,619	11.381	12.150
Age at admission (years)						
Mean	64.3	71.9	66.1	66.9	70.2	74.7
Standard deviation	12.3	11.8	11.6	13.9	12.3	13.1
Previous hospitalization for: cardiovascular disease	17.8	18.5	28.9	25.0	21.4	20.0
- ischemic heart disease	8.1	7.3	13.6	8.6	8.0	5.1
- acute myocardial infarction	0.0	0.0	3.7	3.2	3.2	1.8
- stroke	1.5	1.4	4.6	3.3	0.0	0.0
- peripheral arterial disease	3.1	2.6	0.0	0.0	4.1	2.5
- heart failure	2.1	3.1	3.9	3.5	2.9	3.6
- other cardiovascular disease	6.3	7.5	14.0	15.2	11.6	13.4
diabetes mellitus	6.7	7.2	10.8	13.3	11.1	13.7
Type of hospital (%) - academic	6.2	5.7	11.2	7.8	11.0	9.2
Length of stay (days)	10	11	4	5	22	27
Origin (%) -native Dutch	89.3	90.0	91.5	89.5	89.9	88.9

# Cohort is based on patients first hospitalized for AMI in 1995, † Cohort is based on patients first hospitalized for PAD in 1997 or 2000

‡ Cohort is based on patients first hospitalized for ischemic stroke in 1997 or 2000

### 28-day case fatality

In men, the 28-day case fatality after AMI, PAD and ischemic stroke was 12.7%, 2.4% and 21.0%, respectively. In women this was 20.9%, 3.5% and 23.8% (Table 2). There were large variations in 28-day case fatality in the different age groups (Table 2). The risk of death in AMI patients was much higher compared to PAD patients (men: RR 5.57; 95%CI 4.23 to 7.32, women: RR 6.64; 95%CI 4.99 to 8.84) and lower compared to ischemic stroke patients (Table 3). In PAD, AMI and ischemic stroke patients the overall risk of death was higher for women. However, in the AMI group there were no longer differences in risk of death between men and women after adjustment for age (Table 4).

**Table 2:** Mortality risk at 28 days, 1 year and 5 years after a first hospital admission for acute myocardial infarction (AMI), peripheral arterial disease in the lower extremities (PAD) and ischemic stroke, by age and gender

		Acute myocardial infarction				PAD				Ischemic stroke			
	Age	No. of men	No. of women	Men	Women	No. of men	No. of women	Men	Women	No. of men	No. of women	Men	Women
				% deaths	% deaths			% deaths	% deaths			% deaths	% deaths
28-day case fatality	< 55	3,424	656	4.1	5.8	478	339	-	-	1,336	1,145	6.8	7.5
	55-74	8,027	3,285	10.6	12.8	1,454	778	1.8	1.4	5,549	3,185	14.3	17.2
	75-84	2,563	2,346	26.3	29.8	531	385	5.3	5.7	3501	4739	29.2	25.6
	85+	449	815	38.8	40.0	76	117	18.4	18.8	995	2481	47.8	42.3
	Total	14,463	7,102	12.7	20.9	2,539	1,619	2.4	3.5	11,381	12,150	21.0	23.8
	ASR*			6.0	8.2			0.5	0.5			7.6	9.0
1-year mortality	< 55	3,424	656	5.5	7.3	478	339	2.5	1.5	1,336	1,145	8.9	8.9
	55-74	8,027	3,285	15.3	17.9	1,454	778	7.9 18.6	6.3	5,549	3,185	20.9	20.1
	75-84	2,563	2,346	37.4	39.1	531	385	47.4	16.6	3501	4739	42.2	36.4
	85+	449	815	56.3	55.6	76	117	10.3	41.0	995	2481	61.7	59.2
	Total	14,463	7,102	18.2	28.2	2,539	1,619	3.8	10.4	11,381	12,150	29.6	33.4
	ASR*			7.9	9.9				2.1			9.8	11.3
5-years mortality	< 55	3,424	656	9.4	11.6	478	339	8.4	5.0	1,336	1,145	14.7	12.0
	55-74	8,027	3,285	29.0	30.0	1,454	778	27.7	21.2	5,549	3,185	37.6	35.3
	75-84	2,563	2,346	63.2	60.9	531	385	52.7	45.2	3501	4739	68.2	61.1
	85+	449	815	85.5	81.6	76	117	84.2	74.4	995	2481	86.7	83.5
	Total	14,463	7,102	32.2	44.5	2,539	1,619	31.0	27.4	11,381	12,150	48.6	53.0
	ASR*			13.7	15.9			13.2	7.2			17.1	15.7

PAD: peripheral arterial disease of the lower extremities

*ASR: age-standardized rates (ASR) adjusted to European Standard

- Number of deaths < 3

**Table 3:** 28-day, 1-year and 5-year risk of death after hospital admission for acute myocardial infarction (AMI) compared to ischemic stroke or lower extremity arterial disease (PAD)

		AMI vs PAD			AMI vs ischemic stroke		
		RR (95%CI)*			RR (95%CI)**		
		28 days	1 year	5 years	28 days	1 year	5 years
Men	< 55	-	2.20 (1.24-3.91)	1.13 (0.82-1.54)	0.60 (0.43-0.83)	0.62 (0.50-0.77)	0.64 (0.54-0.76)
	55-74	9.62 (5.78-16.02)	1.43 (1.22-1.68)	1.04 (0.95-1.14)	0.74 (0.67-0.82)	0.73 (0.68-0.78)	0.77 (0.73-0.81)
	75-84	4.99 (3.39-7.36)	2.00 (1.67-2.41)	1.20 (1.10-1.31)	0.90 (0.84-0.97)	0.89 (0.83-0.94)	0.93 (0.89-0.96)
	85+	2.10 (1.21-3.65)	1.19 (0.93-1.53)	1.02 (0.91-1.13)	0.81 (0.73-0.90)	0.91 (0.83-1.00)	0.99 (0.94-1.03)
	Total	5.57 (4.23-7.32)	1.76 (1.56-1.98)	1.04 (0.98-1.11)	0.61 (0.57-0.64)	0.61 (0.59-0.64)	0.66 (0.64-0.68)
Women	< 55	-	4.96 (1.99-12.35)	2.31 (1.39-3.84)	0.77 (0.57-1.05)	0.82 (0.59-1.14)	0.97 (0.74-1.26)
	55-74	14.28 (6.68-30.52)	2.84 (2.14-3.76)	1.42 (1.23-1.64)	0.75 (0.66-0.84)	0.75 (0.68-0.82)	0.72 (0.67-0.76)
	75-84	5.22 (3.38-8.06)	2.35 (1.87-2.96)	1.35 (1.20-1.51)	1.17 (1.09-1.24)	1.07 (1.01-1.14)	1.00 (0.96-1.04)
	85+	2.13 (1.37-3.29)	1.35 (1.08-1.70)	1.10 (0.98-1.23)	0.95 (0.89-1.01)	0.94 (0.88-1.01)	0.98 (0.94-1.01)
	Total	6.64 (4.99-8.84)	2.75 (2.37-3.20)	1.62 (1.49-1.77)	0.83 (0.80-0.88)	0.80 (0.77-0.84)	0.80 (0.77-0.82)

* Relative risk in patient with AMI compared to patient with PAD

** Relative risk in patient with AMI compared to patient with Ischemic stroke

**Table 4:** Gender differences in short-term and long-term mortality after first hospital admission in the Netherlands for acute myocardial infarction (AMI), peripheral arterial disease in the lower extremities (PAD) and ischemic stroke

	AMI	PAD	Ischemic stroke
	HR (95%CI)*	HR (95%CI)*	HR (95%CI)*
Crude			
28 day	0.60 (0.56-0.64)	0.67 (0.47-0.96)	0.88 (0.83-0.92)
1 year	0.62 (0.58-0.65)	0.90 (0.81-1.20)	0.87 (0.83-0.91)
5 years	0.66 (0.63-0.69)	1.15 (1.03-1.29)	0.88 (0.85-0.92)
Adjusted†			
28 days	0.91 (0.85-0.98)	0.88 (0.61-1.28)	1.11 (1.05-1.18)
1 year	0.96 (0.91-1.02)	1.19 (0.98-1.45)	1.14 (1.08-1.19)
5 years	1.06 (1.02-1.12)	1.35 (1.19-1.51)	1.18 (1.14-1.23)

* Hazard ratio in men compared to women

† Adjusted for age

### One-year mortality

Compared to 28-day case fatality, the differences in risk of death between AMI and PAD patients reduced in all age-sex-groups (Table 3). In PAD, AMI and ischemic stroke patients the overall risk of death was higher for women. However, after adjustment for age there were no differences in risk of death between men and women (Table 4).

In men, the 1-year risk of death among patients who survive for at least 28 days after first admission for AMI, PAD and ischemic stroke was 6.2%, 9.0% and 10.9%, respectively. In women this was 9.2%, 7.1% and 13.0% (Table 5).

**Table 5:** Mortality among patients, who survive for at least 28 days after first hospital admission for acute myocardial infarction (AMI), peripheral arterial disease in the lower extremities (PAD), or ischemic stroke, by age and gender

		Acute myocardial infarction				PAD				Ischemic stroke			
	Age	No. of men	No. of women	Men	Women	No. of men	No. of women	Men	Women	No. of men	No. of women	Men	Women
				% deaths	% deaths			% deaths	% deaths			% deaths	% deaths
1-year mortality	< 55	3,284	618	1.5	1.7	224	335	5.3	0.9	1,245	1,059	2,3	1,5
	55-74	7,177	2,863	5.2	5,7	1,436	769	6.7	5.2	4,754	2,941	7,7	7,3
	75-84	1,888	1,646	15.0	13.2	503	363	14.1	11.6	2,478	3,526	18.4	14.6
	85+	275	489	28.7	26.0	62	95	35.5	27.4	519	1,432	26.6	29.3
	Total	12,624	5,616	6.2	9,2	2,225	1,532	9.0	7.1	8,996	8,958	10.9	13.0
5-year mortality	< 55	3,284	618	5.6	6.1	224	335	8.4	3.9	1,245	1,059	8,5	4,8
	55-74	7,177	2,863	20.5	19.7	1,436	769	26.8	20.2	4,754	2,941	27,1	26,8
	75-84	1,888	1,646	50.1	44.3	503	363	49.9	41.9	2,478	3,526	55.0	47.7
	85+	275	489	76.4	69.3	62	95	80.6	68.4	519	1,432	74.6	71.8
	Total	12,624	5,616	22.3	29.7	2,225	1,532	22.9	24.7	8,996	8,958	19.5	39.6

PAD: peripheral arterial disease of the lower extremities

### Five-year mortality

**Figure 2:**
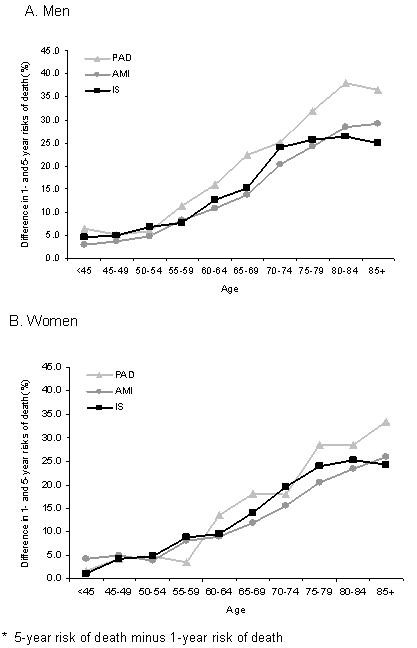
The difference* between 1-year and 5-year risk of death (%) after first hospital admission for acute myocardial infarction (AMI), peripheral arterial disease of the lower extremities (PAD) or ischemic stroke (IS), by age.

In men, the 5-year risk of death after AMI, PAD and ischemic stroke was 32.2%, 31.0% and 48.6%, respectively. In women this was 44.5%, 27.4% and 53.0% (Table 2). Although 5-year risk of death was the highest for ischemic stroke patients, the difference between 1-year and 5-year mortality risks among PAD patients sharply rises and clearly is the highest, especially among male PAD patients (Figure 2). In AMI and ischemic stroke patients the overall risk of death was higher for women. After adjustment for age the risk of death was higher in men than in women (age-adjusted hazard ratio for ischemic stroke, AMI, and PAD: 1.18; 95%CI 1.14 to 1.23, 1.06; 95%CI 1.02 to 1.12, 1.35; 95%CI 1.19 to 1.51 respectively) (Table 4).

In men, the 5-year risk of death among patients who survive for at least 28 days after first admission for AMI, PAD and ischemic stroke was 22.3%, 22.9% and 19.5%, respectively. In women this was 29.7%, 24.7% and 39.6% (Table 5).

### Risk of death for patients without previous admissions for CVD

Differences between PAD, AMI and ischemic stroke patients in the presence of comorbidity may affect relative risk of death. However, we repeated the analysis, and computed 28-day case fatality, 1-year and long-term (5-year) mortality by age and gender according to the actuarial life table method, excluding those patients with previous admissions for cardiovascular disease (CVD). Approximately 18% of AMI patients, 21% of ischemic stroke patients and 28% of PAD patients were excluded. The relative risks of death between the groups remained similar, except for 28-day case fatality, were relative risk for AMI compared to PAD patients increased (data not shown).

## Discussion

The present study shows that the dynamics of mortality over follow-up time clearly differ between patients first hospitalized for atherosclerotic diseases located at different vascular beds. Short-term mortality is the highest for ischemic stroke patients and the lowest for PAD patients. However, the risk of death increases considerably over follow-up time for PAD patients, and 5 years after first hospital admission the differences in risks of death between AMI- and PAD patients and between AMI- and ischemic stroke patients have largely disappeared.

Patients hospitalized for ischemic stroke have the highest risk of death compared to patients hospitalized for AMI or PAD. This finding was in agreement with the study of Caro et al. [8], were they reported crude five-year death rates of 33.2% and 41.8% for patients diagnosed with PAD and stroke respectively. Furthermore, they reported lower 5-year mortality risk for AMI patients (26.6%) than for PAD patients. For women, this finding is in agreement. For men, however we observed similar risks of death between AMI and PAD patients.

The higher age of ischemic stroke patients may have affected risk of death but cannot fully explain the higher risk of death, as the age-standardized mortality rates were also higher for the ischemic stroke patients (Table 2).

The heart and the brains are vital organs, and disturbance of the functioning of these organs may be reflected in the high case-fatality among patients first hospitalized for AMI and ischemic stroke. It has been suggested that apart from the symptomatic atherosclerotic lesion, multiple stable or unstable lesions are present in the whole arterial vessel tree, predicting the patient's prognosis [15]. In addition, it has been suggested that atherosclerotic lesions occur predominantly in the large vessels first, with distal lesion occurring thereafter [16]. It is therefore likely that patients first hospitalized for PAD have atherosclerotic lesions in the whole arterial vessel tree. Our observation that PAD patients had more previous admissions for cardiovascular diseases than the AMI and ischemic stroke patients supports this thought. The association of PAD with future cardiovascular disease events and CVD and total mortality has been demonstrated in multiple studies [17] and underlines the importance of secondary prevention among PAD patients, though PAD patients tend to be under-treated compared to patients with other manifestations of atherosclerosis, and it would appear that cardiovascular risk factor management for prevention in PAD patients is very modest [18,19]. Therefore, clinicians should be aware of the dynamics of mortality over follow-up time for patients admitted for different manifestations of atherosclerosis, in order to provide optimal secondary prevention treatment.

In this study higher age-adjusted 5-year risk of death after admission for AMI, PAD and ischemic stroke was observed for men. This is in agreement with findings from other studies for PAD and ischemic stroke patients [20,21]. Nevertheless, some factors that may be related to the patient's prognosis were differently distributed among men and women. Women were known to be older, have more diabetes and had a longer mean length of stay, while men were admitted to academic hospitals more often and had more previous admissions for ischemic stroke. Furthermore, other predictors for long-term risk of death may have been unequally distributed among men and women in the present study, thereby affecting the risk of death. For example, the prevalence of smoking, which is a risk factor for progression of atherosclerosis [8], is generally higher among men [22,23]. Smoking reduces survival in patients with atherosclerosis [24], and therefore smoking behavior might affect our results through over-estimation of the gender differences in mortality risk. Unfortunately, our study does not permit an in-depth evaluation of these estimates, since no information was available for most of the known predictors for prognosis following ischemic stroke, AMI and PAD.

## Strengths and limitations

The strength of our study is the large size of the cohort, obtained from usual care, with a large age range and information on both men and women. Even though the validity of national registers has been questioned, several studies have shown that for the Netherlands the validity is adequate [25,26] and a high validity of the linkage between the registers has been demonstrated [26,27]. Furthermore, for ICD-9 codes 410, 434 and 436, the positive predictive values have been shown to be acceptable [28-30].

To appreciate the findings, the following needs consideration. The limitation of previous admissions to a maximum of 6 years may have resulted in some "first-time" AMI, PAD or ischemic stroke patients (especially those older than 65 years) who were actually returning patients. The inclusion of returning patients may cause overestimation of absolute mortality rates (as returning patients are usually more severe patients), but not affect the difference between men and women, as there is no difference in return rate between men and women [31-33].

Mortality risks presented in this study are based on AMI patients hospitalized in 1995 and stroke and PAD patients hospitalized in 1997/2000. However there is a substantial change in the way patients are treated now a day (especially AMI patients) and as a result of that current mortality risks may be lower.

Finally, the number of PAD patients was considerable smaller than the number of AMI and ischemic stroke patients. This is due to the fact that we decided to use ICD-9 code 443.9 for the identification of patients with peripheral arterial disease of the lower extremities (this code is described as peripheral vascular disease unspecified, intermittent claudication not otherwise specified). The PAD cohort would be much larger if we also used ICD-9 code 440.2 (atherosclerosis of the extremities), ICD-9 code 444.22 (arterial embolism or thrombosis of the lower extremities) and ICD-9 code 785.4 (gangrene). However, the PAD cohort would also become a case mix of patients with large differences in severity, symptoms and prognosis. Therefore only patients with ICD-9 code 443.9, representing moderate/severe claudication patients (Fontaine stage IIb, III and IV respectively) were included in this study. The ICD-9 codes 440.2, 444.22 and 785.4 may represent the more severe forms of the disease compared to moderate/severe claudication patients. Therefore, the risk of death after admission among these PAD patients is probably higher than observed risk of death presented in this study.

## Conclusions

The present study shows that the dynamics of mortality over follow-up time clearly differ between patients first hospitalized for atherosclerotic diseases located at different vascular beds. Short-term mortality is the highest in ischemic stroke patients and the lowest for PAD patients. However, the risk of death increases considerably over follow-up time for PAD patients, and 5 years after first hospital admission the differences in risks of death between AMI- and PAD patients and between AMI- and ischemic stroke patients have largely disappeared.

Clinicians should be aware of the dynamics of mortality over follow-up time for patients admitted for different manifestations of atherosclerosis, in order to provide optimal secondary prevention treatment.

## Competing interests

The authors declare that they have no competing interests.

## Authors' contributions

IV performed the statistical analysis and drafted the manuscript. ID participated in the design of this study and commented the draft. DG conceived of the study and commented the draft. MB conceived of the study, and participated in its design and coordination. All authors read and approved the final manuscript.

## Pre-publication history

The pre-publication history for this paper can be accessed here:

http://www.biomedcentral.com/1471-2261/10/57/prepub
